# Re-engineering the Cypriot healthcare service system

**DOI:** 10.1186/s12913-020-5048-3

**Published:** 2020-04-07

**Authors:** Elena Pallari, George Samoutis, Anthony Rudd

**Affiliations:** 1 University College London, Medical Research Council Clinical Trials Unit, 90 High Holborn, London, WC1V 6LJ UK; 2 King’s College London Centre for Implementation Science, Institute of Institute of Psychiatry, Psychology & Neuroscience, Health Service & Population Research Department, De Crespigny Park, Denmark Hill, London, SE5 8AF UK; 3 Centre for Primary Care and Population Health, St George’s, University of London Medical School at University of Nicosia, 21 Ilia Papakyriakou Street, Engomi, P.O. Box 24005, 1700 Nicosia, Cyprus; 4 School of Population Health and Environmental Sciences, King’s College London, London, UK; 5 National Institute for Health Research (NIHR) Biomedical Research Centre, Guy’s and St Thomas’ NHS Foundation Trust and King’s College London, London, UK

**Keywords:** Cyprus, Healthcare system, Re-engineering, Transformation, Quality improvement

## Abstract

**Background:**

The Cypriot healthcare system has undergone a number of major transformations since the induction of the Republic of Cyprus in the European Union over 10 years ago. Currently Cyprus is undergoing a major reform, namely the introduction of a primary care driven national healthcare system. The aim of the study was to assess the existing state of training, support, quality, guidelines and infrastructure towards a better healthcare system in Cyprus.

**Methods:**

This is a mixed-methods study combining statistical data until October 2016 and workshop discussions delivered in Cyprus in November 2015. We used anonymised data provided: (1a) by the Cyprus Medical Association of all registered medical doctors up to October 2016; (1b); by the Ministry of Health (MoH) Health Monitoring Unit up to October 2016; (2) during a workshop organised with representatives from the Royal College of Physicians, the European Commission and the Health Insurance Organization.

**Results:**

The gender ratio of men over women is disproportionate, with over 85% of the medical doctors undertaking their training in Greece, Eastern Europe and neighbouring countries, while the current record does not hold a relevant specialty information for 4 out of 10 doctors. The results show lack of statutory inspection systems, application of revalidation principles or implementation of peer-review clinical services on the island. There are eight proposed recommendations made by the workshop participants towards the transformation of the Cypriot healthcare system and the development of the Cyprus Quality Improvement Institute. These are aimed at addressing gaps in quality of care, adherence to clinical guidelines and implementation of audits, development of doctors’ revalidation and peer-review of clinical services, accreditation of service implementation, establishment of a statutory inspection system as well as the set-up of an incentives program as part of the general healthcare system (GHS) of Cyprus.

**Conclusions:**

Current efforts for the implementation of the new GHS in Cyprus call for adequate training and support of the medical workforce, transparent and safer quality of care provision through the implementation of clinical guidelines and capacity-building infrastructure.

## Background

Re-engineering a healthcare system refers to the process of strategic choice and change within a hospital setting to transform process delivery and performance [1]. Following the example of the re-engineering of the Leicester Royal Infirmary, in becoming one of the largest teaching hospitals in the UK [1], we have attempted to assess the needs of the Cypriot healthcare system and suggest ways of contributing to its re-engineering. Cyprus is currently undergoing major transformation in establishing the new universal coverage General Healthcare System (GHS). The economic system underwent systemic changes and fluctuations and there is still work in progress towards a transformative Cypriot GHS that will provide universal coverage to the Cypriot population through the merging of public and private health resources.

In light of these reforms, we set to investigate the current capacity of the Cypriot healthcare to gather evidence-based suggestions and recommendations for the future reconfiguration of the Cypriot healthcare infrastructure in two ways: (1a) To understand the gender ratio and country of study of the current medical workforce registered on the official record; (1b) To establish the percentage attribution between the different medical specialties and whether this meets the needs of the Cypriot population; and (2) To establish a baseline on the current state of clinical guidelines and clinical audit development in Cyprus.

**Fig. 1:**
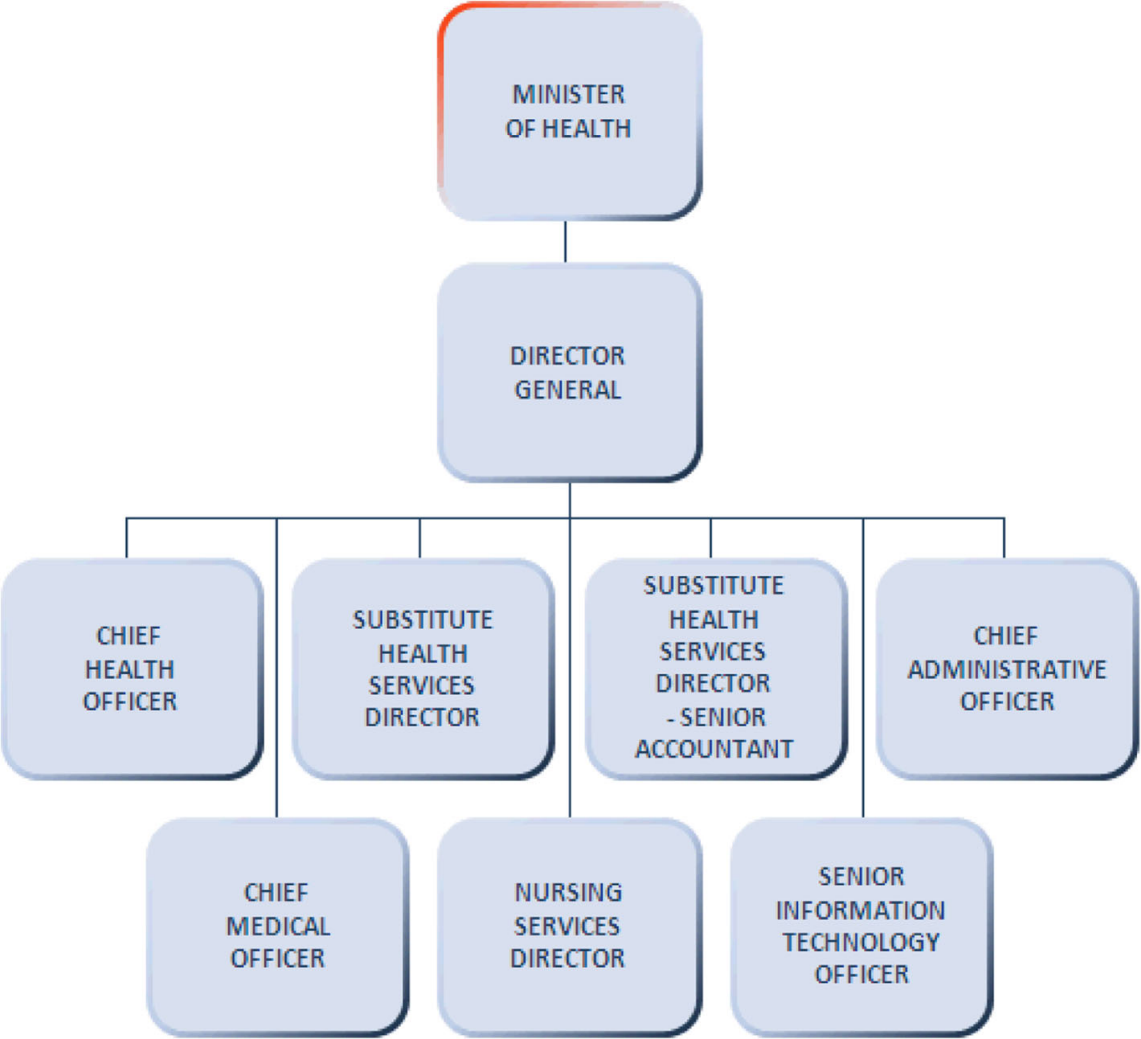
The organisational chart of the Ministry of Health in Cyprus [14]

In Cyprus, the existing political structure prior to the implementation of the new GHS was made up of 11 ministries separated into different departments providing specific services within the Cypriot Government [2] with the Ministry of Health (MoH) being responsible for the health care system of the island. For example, the MoH has been responsible for the formulation of national health policies, the coordination of health care standards, the promotion of the execution of relevant legislation, and the regulation of the activities of both the private and the public sector. In particular, MoH has been in charge of 10 Departments/services, specifically the Nursing Services [3], Purchasing and Supply Directorate [4], European Coordination Sector [5], Health Monitoring Unit [6], IT Unit [7], Blood Bank [8], Medical & Public Health Services [9], Pharmaceutical Services [10], Dental Services [11], Mental Health Services [12] and State General Laboratory [13]. The structure of the Ministry of Health in Cyprus involved Directors and Departments, with Heads, Senior Officers and Professional and Technical staff at each level as shown in Fig. 1.

**Fig. 2:**
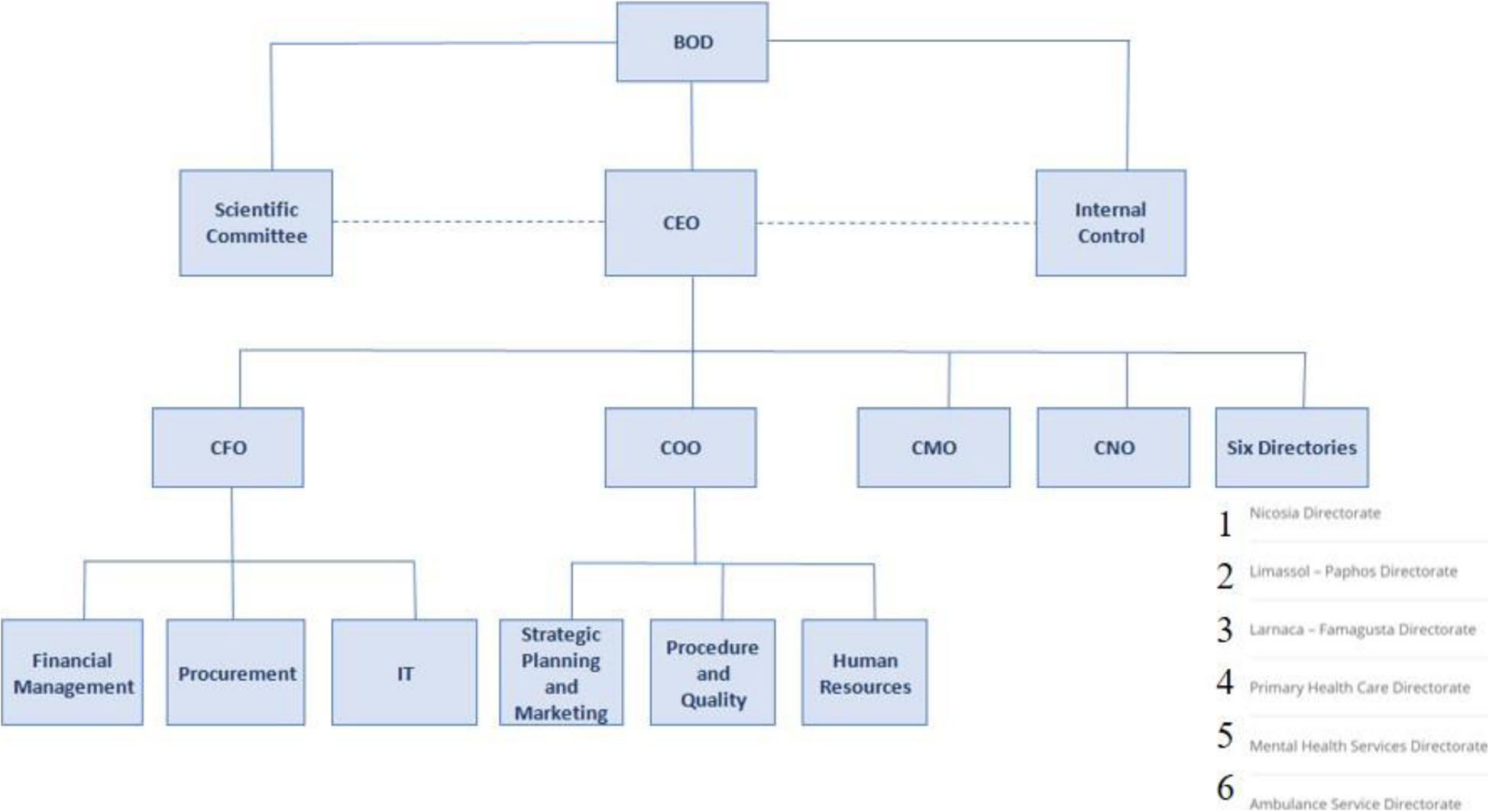
The organisational chart of the State Health Services Organisation (SHSO) regarding the new GHS implementation [adapted from [18]]

This study is focused on the development and implementation of an evidence-based quality improvement (QI) system to be able to improve outcomes both in terms of reduced mortality and disability. The proposed suggestions aim to reduce the quality problems that the Cypriot patients are encountering, lower financial cost, eliminate resource wastage and prepare for the transition to a high quality, universal coverage healthcare system. The State Health Services Organisation (SHSO) mission is to provide patient-centred services to the Cypriot population through a strategy of integrated services [15]. The Health Insurance Organization (HIO) was established in 2001 as the executive authority with the sole purpose of implementing the Global System of Health in Cyprus under the Law No 89(I) 2001 [16, 17]. The structure of the new Cypriot GHS is shown in Fig. 2. The transferred services are under the new system, *ie.* the GHS provide access to the Cypriot population to: personal doctors, outpatient specialists, labs, pharmacies (including medical devices and supplies), nurses, midwives and allied health services, allied health professionals, inpatient healthcare services, dentists, palliative care, rehabilitation care, home care, ambulance service, accident and emergency departments (A&Es) [19].

## Methods

**Fig. 3:**
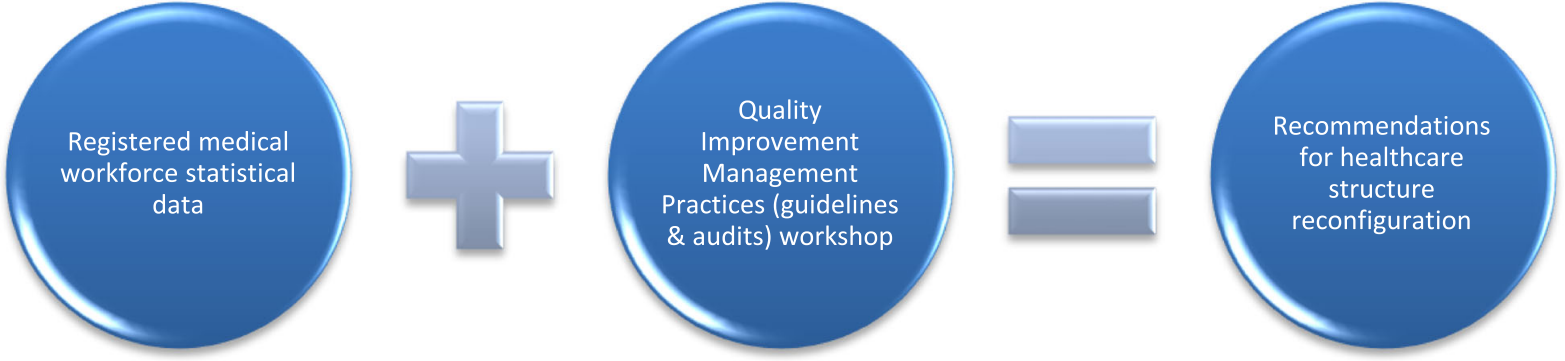
The methodology

This is a mixed-methods study combining statistical data until October 2016 and workshop discussions held in Cyprus in November 2015, schematically shown in Fig. 3. We used anonymised data provided: (1a) by the Cyprus Medical Association of all registered medical doctors up to October 2016; (1b); by the MoH Health Monitoring Unit (HMU) up to October 2016. The rationale of the proposed methodology was to explore any gaps in the existing system of health service provision through the CMA and HMU and assess the potential to fully support the implementation of the new GHS. Secondly, we used data collected during a workshop organised with representatives from the Royal College of Physicians in London, the European Commission (EC) and the HIO. The purpose of this part of the work was to provide an overview and consensus of expert advice and input from a range of key stakeholders towards an informed decision-making to fully support the sustainability of the GHS.

In 2014, a decision was undertaken to collaborate with NICE (UK) and HQIP (UK) in order to develop local expertise on clinical guidelines and audit: the projects were undertaken with the funding support of the European Commission (through the Support Group for Cyprus). Working groups were set up to develop clinical practice guidelines (CPGs) and standard operating procedures, including protocols for laboratory tests, for conditions with high volume and value. The working groups cooperated with physicians from both the public and the private sector to achieve this. Once finalised, the guidelines were handed over to and presented to all general practitioners and clinical laboratories personnel in a series of educational meetings across Cyprus. The working groups reported to senior management of both the MoH and the HIO with a number of conclusions/ recommendations, among which the need for implementation of the guidelines in IT systems, follow-up, revision and elaboration of more guidelines became apparent as imperative. Therefore, the need to “institutionalize” the process has arisen, *ie*. having a system in place on a permanent basis, with a mandate so that team members are allowed to dedicate the appropriate working time to the tasks. The pursuit of the initiative with the working groups is useful while establishing the institutionalization of the process. This can potentially be applied in other areas and not just guidelines, and therefore, the workshop would allow to explore other areas of priority. We set to “map” the current medical workforce landscape and capacity of the Cypriot healthcare context in two ways to investigate its ability in fully implementing the new universal healthcare system. The focus of the study is solely on medical doctors, as the first approach to assess the current state, due to time and resource restrictions. However, as part of a holistic assessment of future needs following the implementation of the Cypriot GHS, we plan to gather information on nurses, other healthcare professionals and allied professionals such as radiographers and healthcare assistants.

### Medical workforce landscape

The first method was used to gain an understanding of the current medical workforce landscape legally working and available to work on the island. For this purpose, we got permission from the Cyprus Medical Association (CMA) to gain access to anonymised data of all the medical doctors including the gender ratio and country of study. We gained access to data for those registered on the official record until and including the 12th October 2016. We also established the percentage attribution between the different medical specialties and whether this meets the needs of the Cypriot population and performance compared to other EU Member States (EU MS) using published data from the World Health Organization (WHO). The anonymised data were kindly provided by the HMU of the MoH. This method contributed to the mapping of the medical specialties on the island for further investigation of how best a quality improvement system can be developed to support the efforts of the doctors through the newly formed GHS.

### Quality improvement management practices

In November 2015 a workshop was organised and delivered in Cyprus in with representatives (*n* = 96) from the Royal College of Physicians of London, the EC and the HIO. The aim of the workshop was to establish a baseline on the current state of quality improvement management practices including CPGs' development and clinical audit implementation in Cyprus. The proposed suggestions by the workshop participants are enclosed following thematic analysis of the meeting minutes.

### Healthcare structure reconfiguration

We provide an interpretation of the findings regarding the current landscape of the registered medical workforce on the island and state of quality improvement of care delivered by the Cypriot health system. We therefore, offer some recommendations around the healthcare structure reconfiguration that can ultimately affect the successful implementation of the universal Cypriot GHS.

## Results

The presented results span across three levels: (1) the current medical workforce landscape (up to 2016) (2) quality improvement, CPGs development and clinical audit implementation state and (3) healthcare structure reconfiguration propositions to go ahead with the proposed GHS.

### Medical workforce landscape

**Fig. 4:**
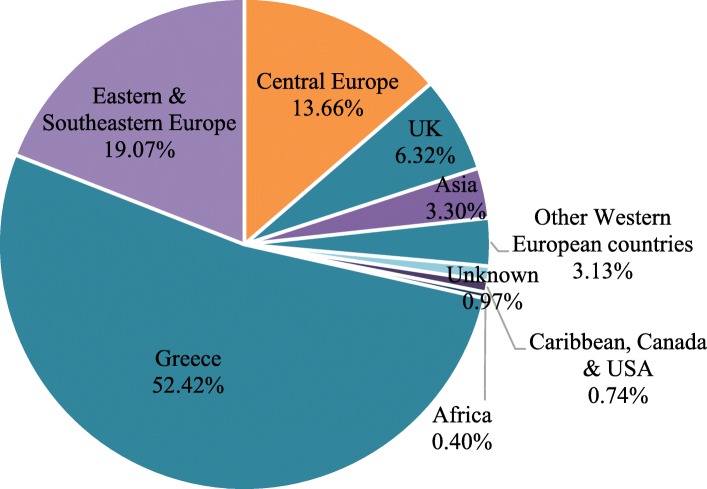
The country of the Cypriot doctors’ education and training up to October 2016 (provided by the Cyprus Medical Association)

From data provided by the CMA, there have been 3651 registered medical doctors of which 2267 are males (62%) and 1384 females (38%) officially registered and legally checked to be able to practice in Cyprus. In Fig. 4, data on the registered doctors’ percentages according to the country of study was provided, with over half attributed to Greece, followed by Eastern and South Eastern Europe countries *eg*. Russia, Bulgaria, Central Europe *eg.* Hungary, Czech Republic, the UK and Asia *eg.* Turkey, Serbia.

**Fig. 5:**
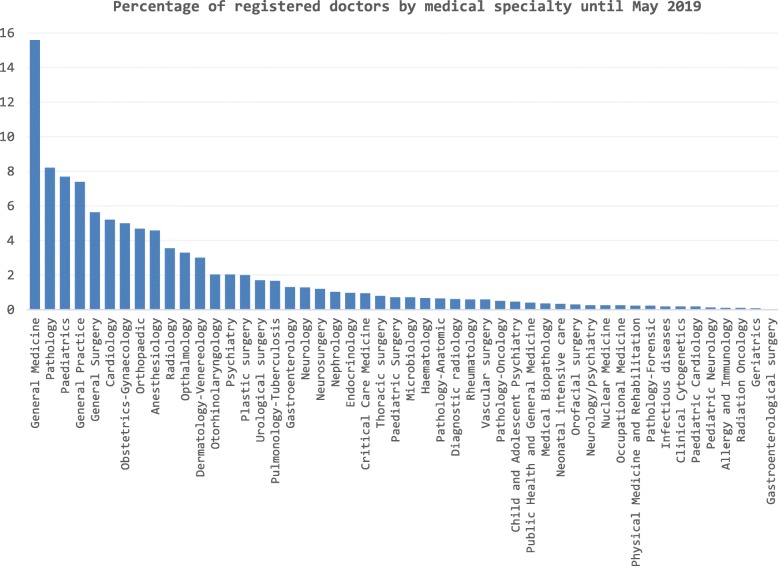
The specialties of registered doctors until May 2019 for Cyprus (provided by the MoH Health Monitoring Unit)

The percentage on registered specialties for the medical doctors in Cyprus is shown in Fig. 5, with 16% having obtained their degree as medical doctors with no further practice of specialisation, while 7% are general practitioners (GPs). Additionally, from information provided by the HMU, also about 4% of registered doctors have a second specialty (data not shown). The results agree with the WHO report published in 2016 [20]. The number of physicians or doctors in Cyprus was the largest relative increase across all EU MS in the WHO study, rising from 297 per 100,000 inhabitants in 2011 to 377 per 100,000 inhabitants in 2016 [20], to 385 for 2019 (up to May 2019, hence not complete data for full year; data provided by the Cyprus Medical Association, see Acknowledgments). However, and based on 948,513 Cyprus controlled-area population for 2019, it is unclear why there’s been a decrease in the number of surgical specialty doctors from over 100 per 100,000 inhabitants to 71 per 100,000 inhabitants. Although the ratio of specialists to generalists in Cyprus was comparable to other EU MS in 2016, this has remained constant for 2019 (3:1).

Perhaps, consideration should be made on increasing transparency in the recording procedures of registering the doctors on the database and addition of a security algorithm that will not allow for registration unless the necessary data and relevant information are captured from the MoH, HMU and the Statistical Service of Cyprus.

### Quality improvement management practices

The discussion from the meeting was transcribed in the form of meeting minutes. These data were then categorised into eight themes as structured during the workshop discussion. The eight thematic sections are shown in Table 1. The workshop participants suggested the adaptation of existing guidelines from other countries as a way of saving time and resources rather than developing de novo guidelines. In this case, the development time would be limited to 3–6 months for each set of guidelines.

**Table 1:** The eight thematic categories of the workshop

**Quality Improvement Management & Practices Workshop**	
1. Clinical practice guidelines (CPGs) development	
2. Clinical audit development and implementation	
3. Capacity building and resource allocation	
4. Revalidation system of clinical services for all clinicians working in Cyprus	
5. Peer-review system set-up for clinical services	
6. Accreditation of services implementation to perform at the highest quality and safety standards	
7. Inspection process implementation to ensure provision of safe and sustainable services	
8. Incentives systems development (rewards, motivation and support) for clinical excellence	

## Discussion

The workshop participants discussed eight key areas to achieve QI management and practice and brainstormed on actionable suggestions to fulfil these.

### Clinical practice guidelines development

It was discussed that the purpose of the development of National Clinical Practice Guidelines for Cyprus are to:

1. Set out the clinical care that is suitable for most patients with a specific condition in Cyprus
2. Improve the quality of clinical care
3. Assess the clinical and cost effectiveness of treatments and ways of managing a condition
4. Set national priorities, reduce bias and minimise conflict, dealing successfully with special interest groups e.g. patient advocate groups, pharmaceutical companies
5. Define quality standards to be able to measure the effectiveness of the guideline introduction through relevant series of clinical audits (this is further explored later on Clinical Audits)

To achieve the above, the following objectives were set:

1. Include a multi-disciplinary team of relevant stakeholders in such an endeavour assembled of healthcare professionals, patients and their carers, Cyprus Ministry of Health (MoH), Cyprus Health Insurance Organization (HIO), Cyprus Scientific Societies, the public, government bodies and the healthcare industry.
2. Use the best available research evidence and expert consensus. The estimated CPG production time is between 18 and 24 months for a standard guideline, and between 11 and 13 months for a short guideline.
3. Use recognised methods that are methodologically and clinically sound and transparent.

Recently, it was demonstrated that CPGs in oncology differ in their underlying evidence-base due to the temporal and geographical discrepancies of cited evidence [21]. More recently, it was shown that Cyprus is lagging behind the development of clinical practice guidelines [22]. Our findings are in agreement with these findings. It was found that clinicians could be supported by the MoH, Scientific Associations and relevant professional bodies like the Cyprus Medical Association, Cyprus Nurses and Midwives Association, Cyprus Federation of Patients and representatives, and other relevant stakeholders to find ways to develop, implement and institutionalise clinical guidelines development in Cyprus. Currently there is no established body or mechanism on the development of CPGs on the island and healthcare professionals usually consult European or UK guidelines. In this effort and in light of the GHS being implemented, healthcare professionals across the different medical departments and hospitals could work collaboratively for the development of hospital guidelines and protocols as they see fit to the needs of the population they serve.

### Clinical audits development and implementation

Cyprus is a relatively small country and the creation of such universal healthcare system to provide access to the nation through excellent services is a very ambitious goal that requires the appropriate support if it is to be successful and sustainable. Cyprus healthcare requires a unified independent service that can run national audits and provide guidance, education and training in clinical audit methodology and QI tools to clinicians and support staff. This is currently lacking. The SHSO oversees the operation of the six directorates (see Fig. 2) covering the Cypriot population: nationally, the capital district, the remaining four districts, as well as primary care, mental health and ambulatory services. In the effort to support the operating hospitals and clinics within each directorate, academic departments providing training to the healthcare workforce have a very important role in this. Key skills required to achieve this goal include clinical decision making, project management, data collection design and analysis, report writing, training, knowledge and experience in QI. The outcome of the clinical audit services should be a series of national audits on conditions identified as priority areas. Each audit will provide ‘state of the nation’ and individual provider organisation reports. Individuals and teams should be recruited in provider organisations with the skills and knowledge to develop local audits and implement changes in practice that lead to sustained improvements.

Draft policy documents have also been produced and include a clinical audit policy setting out the principles, roles, responsibilities and practices which should be followed when auditing clinical practice. It also includes a clinical audit strategy, which describes how the MoH and HIO will implement the policy. Most importantly based on the field work from the NICE/HQIP/RCP/Cyprus projects, it was concluded that it is feasible to implement clinical guidelines and clinical audit in a small country with limited resources, by designing a suitable quality management framework and set up an appropriate independent body to lead the process.

A further role to support this effort should be provided by the Chief Executive and Medical Officers (CEOs and CMOs). A plethora of benefits are expected from the function of this service. Firstly, this organisation or centre can provide the platform for national and local recommendations for improvement and benchmarking across departments and hospitals which can demonstrate areas at which providers are doing well and which require further development. Moreover, it will provide a consistent, unified use of audit methodology supported by a central team and responsive to the needs of clinicians and organisations. Patient involvement can be encouraged and supported. Cross sector audits can be carried out additionally to follow the patient pathway.

### Capacity-building and resource allocation

Service-users have for many years reported problems with the quality of the current healthcare system. The proposed implementation of the Cypriot NHS is thought to provide greater autonomy and better management of the public hospitals, introduction of information technology systems and involvement of the patients and public in developing and designing services. The planned reforms are very ambitious and will face an array of challenges including the need for significant investment and wide political support to be implemented. The autonomisation of the public hospitals started in January 2019 while the first phase of the new GHS started in June 2019 (primary care, laboratories, imaging and outpatient specialists). The final phase of the reforms will be implemented in June 2020 concerning the hospital care and allied healthcare professionals. By then and taking into account that GHS is within the first year of its operation, the imperative milestone of capacity-building and resource allocation should be performed. Through an evaluation of the existing state of performance we can identify unmet needs and plan better on how to meet these. An example is the requirement for investment in lifestyle or behavioural interventions. It was recently shown that Cyprus has the highest relative burden from diabetes in Europe, according to the World Health Organisation, and it was found that the topic however is seriously under-researched [22]. The organisations conducting research on diabetes are University of Nicosia, Nicosia General Hospital and the University of Cyprus followed by the European University, Ygeia Polyclinic and seven other Cypriot institutions. The same situation applies for mental and respiratory conditions. Perhaps, the operating directorates under SHSO can closely collaborate with the relevant funding bodies and research organisations on the island for the set-up of research programs or clinical studies such as using collected data from patient cohorts to inform the better management and treatment of patients suffering from these conditions.

### Development of a revalidation clinical services system for all clinicians working in Cyprus (medical doctors and allied health professionals)

Continuing to practice effectively as a clinician requires the individual to keep up to date with new developments in their field as well as continuing to demonstrate skills in communication, organisation and ethical practice. Revalidation aims to ‘demonstrate that the competence of doctors is acceptable’ and is attracting increasing interest in Europe, drawing on the experiences of the USA, Canada, Australia and New Zealand. However, the practice of revalidation in different countries varies. In its most basic form, it involves participation in continuing medical education (CME), designed to keep physicians up-to-date with clinical developments and medical knowledge. The broader concept of continuing professional development (CPD) includes CME along with the development of personal, social and managerial skills. Responsibility for administering revalidation varies between countries ranging from professional medical bodies to insurers. In the UK, participation in CPD is a condition of employment in the NHS and recently, the Department of Health and Social Care introduced a compulsory system of revalidation including all physicians, in whatever setting they practice. Physicians are required to renew a licence to practise every five years with the process run by the General Medical Council (GMC). In the USA revalidation is provided through a self-regulation assessment, and although not compulsory, the American Board of Physician Specialists (ABPS) requires this for their members. Currently, only Germany, the Netherlands and the UK have formal revalidation systems in place. Informal methods of revalidation exist in Austria, Belgium, France and Spain. These programmes are heavily dependent upon participation in CME as the mechanism to maintain physician competence. Belgium and France also take revalidation a step further by including peer review [23]. A notable transferrable example is the regulation set from the Cyprus Nursing and Midwifery which requires nurses and midwives to hold a valid licence to practice and they must renew it every four years.

Initially, the SHSO should provide a requirement for the revalidation of doctors and this should subsequently be expanded to all health professional (professions allied to medicine). It should be a statutory rather than a voluntary system and have as its core component parts a requirement to complete a minimum of continued professional development per year and an annual process of individual performance review. It is recommended that the CMA runs the scheme. Every five years a portfolio would need to be submitted to the CMA, a 360-degree appraisal from professional colleagues and feedback from patients that the physician had treated. The GMC document ‘Good medical practice’ could form the basis of the revalidation process [24]. No one should be permitted to continue to practice unless they have been revalidated. Another proposal from the workshop was that a cohort of clinicians should be trained to be ‘responsible officers’ able to evaluate the portfolios and be given the responsibility to approve the continued practice of the Cypriot clinicians. There would need to be at least 100 of these responsible officers across the five districts each supervising about 30 colleagues revalidating once every 5 years. However, for the annual appraisals there should be a system where the clinical director of the department in the hospitals and senior primary care physicians take on the task.

### Development of a peer-review clinical services system

A system of invited service review should be established in Cyprus, administered by the CMA. There should be a generic model able to apply at services from all specialities and then smaller services covering the key disease areas *eg.* cardiac, cancer, stroke and maternity. The costs should be borne by the acute providers and/or the insurance companies, but it should be a non-profit making exercise. Clinicians would be asked to volunteer their services and participating in visits should be allowed by their employers, taking place during their normal working hours, and not be formally remunerated but with CPD points offered for the time involved.

Reviewers should be clinicians ideally who have expertise in their field and preferably from services that have been demonstrated to be running effectively. Training should be provided for the reviewers and a structured framework developed for visits. Typically for the stroke peer review scheme the request is made from the clinical service wanting a visit supported by the chief executive. A chair-person should be appointed to lead the visit and a discussion between the chair and the clinical service. Depending on what the key issues to be addressed are, a team should then constitute of physicians, radiologists, emergency care doctors, nurses, therapists and patient representatives. The visit can take place over one or two days. Discussions take place during the visit with all relevant managers and clinicians on the basis that all information is given anonymously. Preliminary feedback can be provided at the end of the visit and a formal report provided within 6 weeks of the visit. Examples of the sort of advice given include suggestions about the staffing or organisation of care, the need for better leadership and improved governance structures. The service requesting the review pays for the costs of running the visit, typically about £10,000. This is an example of a non-profit making scheme.

A good summary of the evidence and process of peer review was published by the Australian commission on Safety and Quality in Health Care in 2013. A scheme based on the best available evidence and principles was established in the UK to provide peer review for stroke services. The core principle is to provide expert advice and support to clinical services trying to implement QI [25].

### Accreditation of services implementation to perform at the highest quality and safety standards

Accreditation in Cyprus healthcare currently is minimal and covers mainly laboratories and few hospitals such as the BOCOC Oncology hospital and the Ygeia private Hospital in Limassol. There is no legal enforcement of accreditation of services. The biggest challenge is the cost that is entailed in the process and the debate around which organisation should cover it, government or the individual hospitals. Cyprus should gradually establish an accreditation process that would ensure continued quality improvement of its healthcare organisations. The design of the accreditation programme should be simple with minimum cost avoiding bureaucratic processes. The design of the programme needs to involve the representatives of the Cyprus healthcare organisations from the beginning of the process. In this effort, patients will be represented by the National Health Insurance Organisation Board of Directors and have an important role in this transitioning phase of a game-changing National Health Service [26].

Quality management is very limited and there is enormous variation in the care delivered. There is no dedicated unit, organisation or team that is overseeing the quality of care that is delivered. Patients are not yet involved in any QI activities [27]. Accreditation is a professionally-led supportive process involving self-assessment and external peer assessment to estimate the quality of clinical services in relation to established quality standards and promote continuous quality improvement. Based on discussion with all the stakeholders an immediate plan should be in place all public hospitals and the large private clinics should follow the best practices done by the aforementioned referred hospitals (BOCOC and Ygeia polyclinic).

### Statutory inspection system set-up

Developing and implementing a statutory inspection system (SIS) of hospitals, primary care clinics, nursing homes and all other facilities that provide health care, whether public or private, is a key component of delivering a safe and effective health service that the public can feel confident is safe and effective. To date there are audits taking place in hospitals, both from internal and external auditors. However, having a system in place that is mandatory and centralised can ensure that best practices are shared and lessons learnt. Such a centralised system will not only identify failing services that need support but also monitor the financial viability of services and provide a stimulus for services to address problems. Knowing that if they fail to do so the inspectors will impose formal sanctions on hospital departments and even potentially take over the running of the service or limit its activity will be a further incentive for maintaining excellent standards.

The Care Quality Commission (CQC) in the UK is probably the most comprehensive health and social care inspection system that has been established anywhere in the world. The organisation does in-depth inspection of all hospitals, primary care clinics and care homes on a regular basis looking at all aspects of clinical care and management. The CQC is however a very large organisation and inspections are conducted often by enormous teams of over 100 people. It is therefore a very expensive model that would not be appropriate for direct transposition to Cyprus. Nevertheless, the core elements which include regular assessment and interim monitoring of quality data to determine the frequency of inspections, involvement of lay representatives in the visits and the range of areas that are considered, both clinical, managerial and financial would be appropriate for implementation in Cyprus. The MoH should take concerted efforts in order to support the establishment of an organisation similar to the UK CQC (see healthcare structure reconfiguration) as an independent organisation that will carry out the SIS.

### Incentives systems development

The new systems that are being proposed for QI in Cyprus are heavily dependent on harnessing the expertise and goodwill of clinicians working within the health services. There is not going to be enough money to fund large numbers of permanent staff to deliver the schemes outlined above and so ways are needed to find, encourage and support clinicians to seek working at a national level on QI that is both satisfying personally but also seen as a form of self-improvement. This system can reward clinicians financially if they put significant time and effort into advancing clinical care. Most of these schemes would not require much additional resource except for the clinical excellence scheme; and that does not need to start at a high level unless it was decided that this might be one way of attracting clinicians currently working in the private sector to devote more time to the public health service. Possible options that could be considered are briefly described below. There is no specific incentives system in place to date, and concerted efforts should be initiated from all stakeholders (MoH, SHSO, HIO, CMA, Patients Association) in order to design and implement a specific incentive scheme based on the following UK-examples:

#### Clinical excellence rewards

For clinicians who contribute significantly over and above their contractual responsibilities the opportunity could be provided to apply for financial awards to supplement their salaries. In the UK the system is arranged with a series of points from 1 to 12. In 2010 1–5 awards were each worth an extra £2950 a year, and levels 6 to 9 awards were each worth an extra £5900 a year. Level 9 provides an extra £35,400 a year, 10 is worth about £46,000, 11 is worth about £58,300, and 12 just under £75,800. Awards are pensionable. It would be unusual for a consultant to begin accumulating points less than 10 years after appointment and the proportion of doctors receiving the higher-level awards is very small (in the UK 0.6% of consultants hold a platinum award). Clearly the precise level of remuneration would be decided by the MoH.

#### Plans for building time into jobs for quality improvement

Every clinician working within the public health system should have a job plan agreed by their line manager each year. It should be expected that most clinicians would be given at least one session (half a day) per week for quality improvement work including audit, participation in a national quality improvement initiative in some way.

#### Support for the development of leaders

High quality clinical leadership is central to quality improvement. Some leaders develop naturally, others can be developed with training and mentoring. It is suggested that leadership courses are set-up, and appropriate clinicians encouraged to participate. Ideally, potential leaders should be identified early in their careers and this aspect of their training should be integrated into their clinical experience. Leaders should not be chosen on the basis of their seniority, rather on the basis of their aptitude for such roles. The traditional medical hierarchy of the doctors being the leaders and the other clinicians being the followers has no place in a modern health service and the opportunity to lead should be made available to the full range of clinicians.

#### Mentoring

All new consultants and newly appointed staff to other senior clinical roles should be offered the opportunity of having a mentor to support them for the first or second year of their appointments. These would be senior clinicians from their own field of expertise, preferably chosen by the person wanting a mentor. Training should be offered to potential mentors and time allowed in their job plans to undertake this activity.

### Healthcare structure reconfiguration

The workshop participants proposed the development of the CQII (pronounced CQ2) which stands for the Cyprus Quality Improvement Institute, to oversee all the facets of service improvement in light of the new GHS currently in the first phase of its operation. It was suggested that CQII is to be an independent body without direct links to providers, the MoH, SHSO, HIO or health insurance companies (private or public). Through the CQII it was suggested that there would be the need for approximately 10 FTEs (scientists, health economists, administrators, medical adviser) to be recruited initially. Gradually the personnel numbers can be increased as needed. Outsourcing part of its activities could also be an option (biostatistician support *etc.*). The CQII could also obtain support from academic institutions either from Cyprus or abroad. The leadership team and the personnel of the QII should be recruited on a contractual agreement base (they should be independent from the MoH). The executive director of the Institute shall be appointed by the Minister of Health following discussion and agreement from the parliament and shall report directly to them (Minister of Health /parliament). Key stakeholders of CQII shall be the Ministry of Health, Health Insurance Organization, Cyprus Medical Association, Cyprus Nurses and Midwifes Association, Cyprus Patient Association and Medical Academic Institutions in Cyprus.

### Vision of the CQII

The main mandate of the CQII shall be to ensure healthcare services provide people with safe, effective, compassionate, high-quality care and encourage the services to improve. The CQII will work towards implementing in Cyprus a single shared view of quality in healthcare. The role of the CQII shall be to monitor, inspect, support and regulate services to ensure that they meet fundamental standards of quality and safety. The CQII is to promote transparency of the results by publishing the findings and providing recommendations to help policy makers in informed decisions. This is not an easily achieved vision and it will potentially take years to build up and developed successfully. However, the mission of CQII is to support the efforts of healthcare staff and providers to best serve the Cypriot population with the vision of the institute to provide evidence-based recommendations for research and practice. Therefore, we are optimistic that the CQII will be favoured by all political parties, patient associations, providers, professional associations, private and public health insurance organisations and the Ministry of Health to support the New Cyprus National Health Service bill currently being in effect [21].

### Structure of the CQII

Specifically, from the workshop, the following suggestions were made with regards to the role of the established CQII to be on the eight identified priority areas along with the provided resources/ support needed, shown in Table 2. It is important that the proposed service reconfiguration is established in a clear and rigorous way including a legal framework on the daily operations of the Institute. As an independent body, CQII will be supporting the six directorates, the main hospitals, rural hospitals and clinics and private clinics within the GHS.

**Table 2:** Summary of the workshop discussion priority areas, their purpose and resources needed for their implementation

Priority areas	Purpose	Resources
CPGs development	Development of clinical practice guidelines on the key clinical issues that are important in Cyprus	• Oversight committee which will be the committee that will be constituted to lead the Cyprus Quality Improvement Institute (CQII)).
		• Quality improvement leads in each hospital that will coordinate clinical audit, clinical guidelines implementation etc.
		• Expert personnel from the CQII will collect and analyse data and
		• CQII administrator will organise outreach visits and coordinate the development of the reports.
Clinical audit development and implementation	Development and implementation of national audit programmes based on the recommendations in the guidelines	Same as above
Capacity building and resource allocation	Capacity-building and resource allocation	Same as above
Revalidation system of clinical services for all clinicians working in Cyprus	Development of a process for revalidation of all clinicians working in Cyprus	Same as above
Peer-review system set-up for clinical services	Development of a system for peer-review of clinical services	Same as above
Accreditation of Services	Development of a system for accreditation of services	There would need to be a central secretariat support from CQII that will ensure that all doctors practicing is monitored and that the responsible officers maintain their training. CQII shall need Web based tools, systems for collecting patient feedback and for the 360 appraisals.
		• Oversight committee (CQII)
		• A panel of individuals to undertake the visits (clinical, managerial and lay) with a chair who would have responsibility for writing the report.
		• Administrator to organise the visits and coordinate the development of the reports
Inspection process implementation to ensure provision of safe and sustainable services	Implementation of an inspection process to ensure that all health care providers are providing safe and sustainable services	• Run an independent body without direct links to providers, department of health or the insurance companies:
		- CQII Committee to oversee the processes, develop the assessment tools, scrutinise the reports and take responsibility for the decisions about service provision. Should include clinicians, lay people, finance experts and health service managers.
		- There would need to be an administrator of the scheme, planning visits, coordinating the paperwork and report writing
		- A panel of individuals to undertake the visits (clinical, managerial and lay) with a chair who would have responsibility for writing the report
Incentives systems development (rewards, motivation and support) for clinical excellence	Implementation of a process where excellent work by clinicians, additional to normal contractual requirements are rewarded.	Monetary funds for the Clinical Excellence awards. The funding shall come by the government or preferably EU funds (structural etc.). CQII will seek support from external organisations with expertise in quality improvement i.e. from UK organisations such as NICE, Care Quality Commission, Royal College of Physicians etc.

## Conclusions

Cyprus has been planning to reform its healthcare system since 2001 but kept failing to implement the long-awaited National Health Insurance Scheme. This was mainly due to a lack of political will and direction, challenges between the key stakeholders such as the HIO, CMA, Ministry of Finance and in striking a balance between establishing a cost-effective but accessible service to all citizens. Now GHS is undergoing the first phase of implementation until the 30th May 2020. This set milestone will show whether the GHS can deliver universal health services to the Cypriot population. The aim of the GHS is to unify the healthcare services in the public and private sector and establish the tools for quality management such as a universal information technology system. Currently, the new GHS capacity is being tested under the outbreak of the novel coronavirus pandemic (covid19). Perhaps revising the initial set-date for the GHS evaluation and more importantly revising the plans around the re-engineering of the healthcare system may be of better value. At this stage we need to strengthen the GHS so that can truly provide universal health coverage. The aim of the GHS is to unify the healthcare services in the public and private sector and establish the tools for quality management such as a universal information technology system.

This research work demonstrates for the first time, that there is a need for the provision of systematic data recording systems, collaborative approaches and shared decision-making to establish a robust Cypriot healthcare system following discussions on the reconfiguration of its structure. There is no equal gender representation from the medical workforce as it is. This calls for more transparency as to what the newly graduated medical doctors can specialise in, to meet the needs of the population. Additionally, there is no dedicated unit, organisation or team that oversees the quality of care that is delivered on the island. Patients have participated in certain committees such as the Scientific Council of the State Health Services Organisation and contributed in the development of educational programmes for nurses. However, they are not yet involved in any quality improvement activities or co-development of research programmes. Now there is the opportunity through the CQII for a transparent and inclusive system that invites the views of patients, as well as healthcare professionals in creating truly patient-centred processes.

Furthermore, there is no revalidation or peer-review system in place for medical doctors, no statutory inspection procedures in place, while accreditation of services at the hospital level are absent. The media have reported several patient safety issues, medical errors and never events occuring especially in the public sector, causing anxiety and lack of trust in the quality of delivered healthcare services. The proposed actions focus on the development of clinical practice guidelines, and clinical audits development as a remedy for some of these system failures and in setting standards of excellence. The vision and proposed structure of the CQII aims to address these challenges as well as provide capacity-building and resource allocation as needed. Through this study, a methodological approach was taken to provide evidence-based recommendations for the development of a cost-efficient, patient-centric, safe, effective, compassionate, caring, and well led healthcare service in Cyprus.

The current state of affairs in Cyprus involves the implementation of a new general healthcare system. To better support the success and sustainability of the GHS, there is the imperative need for evidence-base changes that support its reconfiguration of services. The opportunity to aid the GHS became evident through an analysis of its healthcare workforce and identification of eight key priority areas. The need for re-engineering the healthcare system is now more immense than ever before, and needs to happen in collaborative partnership if it is to provide trully equal access and delivery of excellent care to the Cypriot population.

## Data Availability

Please see in text (Methods) for specifics on who provided permission and access to data. These data in particular were provided by the Ministry of Health, the Health Monitoring Unit, and the Cyprus Medical Association. There are no other associated data or files to the presented manuscript.

## Abbreviations

CQC: Care Quality Commission
CQII: Cyprus Quality Improvement Institute
GHS: General Healthcare System
HIO: Health Insurance Organization
HMU: Health Monitoring Unit
MoH: Ministry of Health
SHSO: State Health Services Organisation
WHO: World Health Organization
EC: European Commission
CMA: Cyprus Medical Association
